# How effective is a brief educational intervention on prescribing first-line antibiotics in acute cystitis? A quasi-experimental study among general practitioners in Croatia

**DOI:** 10.3325/cmj.2022.63.362

**Published:** 2022-08

**Authors:** Željko Vojvodić, Suzana Mimica

**Affiliations:** 1 Department of Family Medicine, J. J. Strossmayer University of Osijek Faculty of Medicine, Osijek, Croatia; 2 Department of Internal Medicine, J. J. Strossmayer University of Osijek Faculty of Medicine, Osijek, Croatia; 3 University Hospital Centre Osijek, Osijek, Croatia

## Abstract

**Aim:**

To assess the effectiveness of a brief educational intervention on prescribing first-line antibiotics in acute cystitis.

**Methods:**

This quasi-experimental before-after study was conducted over a period of eight months. We collected prescribing data related to urinary tract infections four months before the educational intervention and four months after it. Aggregate data on office visits, diagnoses, and issued prescriptions were collected from each practice’s electronic medical records based on monthly reports.

**Results:**

Overall, 3581 prescriptions were issued: 1717 before and 1864 after the intervention. The total number of prescriptions increased by 8.5%. The use of first-line antibiotics increased by 21.2%, the use of fluoroquinolones decreased by 6.6%, while the use of beta-lactams remained unchanged. After the intervention, nitrofurantoin was the most prescribed first-line antibiotic. The proportion of women who were prescribed first-line antibiotic did not reach the acceptable range (80%-100%) according to the European Surveillance of Antimicrobial Consumption quality indicators. The proportion of fluoroquinolones (17.9%) use was well above the acceptable range (0%-5%).

**Conclusion:**

A brief educational intervention proved to be a useful method in adopting better prescribing habits. Of particular importance is the considerable increase in the use of nitrofurantoin due to its reliable efficacy against multidrug-resistant urinary pathogens.

The treatment of urinary tract infections (UTIs) in primary care abounds with dilemmas, despite their relatively simple and easily recognizable symptoms and the presence of still effective antibiotics and high-quality antimicrobial guidelines. Existing antimicrobial treatment strategies in both inpatient and outpatient settings are becoming less effective, and new and reliable antimicrobial agents are lacking (1). According to Centers for Disease Control and Prevention (CDC) criteria, extended-spectrum beta-lactamase (ESBL)-producing *Enterobacteriales* and multidrug-resistant *Pseudomonas spp* pose a serious antibiotic resistance threat (2). The most important driver of resistance is the excessive use of antibiotics, especially those with a high potential for developing resistance, such as cephalosporins and fluoroquinolones (3). The increase in *E. coli* resistance in the outpatient setting and the ineffectiveness of cephalosporins, fluoroquinolones, and co-trimoxazole necessitate strict control of prescribing practices for the treatment of UTIs in primary care (4-7). According to Croatian multidisciplinary program, the resistance of *E. coli* (the most common cause of UTIs in primary health care) between 2015 and 2019 ranged from 19% and 20% (8).

Antimicrobial treatment of UTIs in Croatia is characterized by a high consumption of fluoroquinolones and beta-lactams (co-amoxiclav and cefuroxime). The consumption of fluoroquinolones has continued to increase over the past decade despite restrictions in prescribing (8). Although national guidelines for antimicrobial therapy of UTIs were issued in 2007 (9), no studies in Croatia assessed the adherence to the guidelines as their primary objective. The guidelines generally emphasize the self-limiting course of uncomplicated cystitis, the empirical use of first-line antibiotics, and the need for a clear diagnostic distinction between uncomplicated disease and complicating factors and comorbidities (9,10). Among first-line antibiotics, nitrofurantoin has played the prominent role, maintaining its efficacy over 70 years of use (11,12).

The guidelines recommend that microbiological culture is unnecessary in uncomplicated cystitis (13), and that the dipstick test and microscopic examination reliably confirm or reject the diagnosis. The negative dipstick test for leukocytes and nitrites has a high negative predictive value because it correlates with a normal microbiological finding in 80%-98.5%of the cases (14,15). The guidelines generally emphasize the pointlessness of urine cultures in uncomplicated UTIs (13) and a high reliability of dipstick tests (14,15). Antimicrobial treatment based solely on clinical symptoms and simple tests, and with a limited number of drugs, is becoming increasingly important in primary care. This is due to the increasing incidence of resistant of *E. coli* isolates in the outpatient setting and the efficacy of nitrofurantoin and fosfomycin against ESBL-producing strains of Enterobacteriaceae (16). In Croatia, recent studies proved the efficacy of fosfomycin against highly resistant urinary pathogens (17,18).

Educational interventions are one of the most common tools used to counteract unfavorable trends in prescribing. They usually target one of a few main objectives, such as: i) harmonizing prescribing practices with guidelines, ii) reducing the frequency of resistant strains in the community, and iii) reducing the use of all antibiotics in general or individual subgroups (19).

This study aimed to evaluate the effectiveness of a brief educational intervention among general practitioners (GPs) in Croatia targeted at increasing the prescription of first-line antibiotics in uncomplicated cystitis and reducing the use of fluoroquinolones. A secondary aim was to assess the quality of prescribing according to the European Surveillance of Antimicrobial Consumption (ESAC) quality indicators for outpatient antimicrobial utilization (20).

## METHODS

This quasi-experimental before-after study conducted from March 2019 to October 2019 involved 42 family-medicine teams. In the first four months of the study (from March to June 2019), we collected data on the usual prescribing practices. The educational material was distributed to GPs by email in late June 2019. To assess the effect of the intervention, in the second part of the research (from July to October 2019), we continued to collect the same data. We also evaluated whether the prescribers understood the main information from the educational intervention.

During the preparatory phase, which started in August 2018, 105 GPs from entire Croatia were contacted by telephone or email, and were sent an introductory letter with an invitation to participate in the study. A total of 42 GPs responded: 34 women and 8 men. Most of the GPs who refused to participate justified their decision by work overload. If we had continued to contact GPs and include new ones, the sample would have been more representative, but this would have significantly exceeded the time provided by the research plan. The sample of physicians who accepted the participation was comparable in demographic and workplace characteristics with the sample of contacted physicians. In addition, physicians from the sample were comparable in demographic characteristics with the population of family physicians in Croatia. They were from all Croatian regions, from both urban and rural areas. Specialists in family medicine (64%) slightly outnumbered non-specialists (36%). Since both of them work in the same circumstances and care for the population with the same demographic characteristics, we considered the existing sample sufficiently representative. In Croatia, most GPs work in individual practices, and therefore all participants in the sample worked in their own practices.

### Data collection

Prescription-based data on antimicrobial use were collected from each practice’s electronic medical record. Data were submitted once a month in aggregated form. All antimicrobial prescriptions related to the diagnoses from N10 to N49 (complicated and uncomplicated infections) according to the 10th revision of the International Classification of Diseases (ICD) were recorded, as well as the number of prescriptions for uncomplicated cystitis (ICD codes N30 – N30.9).

We analyzed the use of ten antimicrobials associated with the diagnoses of interest: nitrofurantoin, fosfomycin, sulfamethoxazole-trimethoprim, amoxicillin-clavulanic acid, norfloxacin, ciprofloxacin, cefuroxime, cephalexin, cefixime, and cefpodoxime, irrespective of whether they were recommended in guidelines or not. GPs recorded every prescription associated with these diagnoses in EMRs (in real time), regardless of whether the UTI was microbiologically confirmed. Diagnoses were most often based on medical history and/or test strips. Diagnosis based on microbiological examination was possible, but in the aggregated data we were unable to determine the share of diagnoses based on urine cultures. Repeated prescriptions of the same or different antibiotic to the single patient within one month were recorded as two separate prescriptions. Because of technical limitations in EMRs, we were unable to accurately determine the time between two prescriptions. Since all personal data were replaced by identification codes (each visit to the GP that resulted with a prescribed antibiotic for UTI was assigned a new code), it could not be determined whether a patient was prescribed twice within a month. Feedback from general practitioners was sent on a monthly basis, and the entire process of data collection and transmission was anonymous.

### Intervention

The material for the educational intervention consisted of one 14-page review-like article in Croatian. The first part of the article provided information on the rationale for choosing first-line antibiotics (nitrofurantoin, fosfomycin, and co-trimoxazole) and on the reliability of medical history and simple tests in distinguishing uncomplicated from complicated cases. In the second part, 17 cases from routine practice were described and corroborated by comments. All information provided in the educational intervention conformed to the relevant European and national guidelines (3,10,11). The importance of defining as clearly as possible the broad clinical context and complicating factors in distinguishing between uncomplicated and complicated infections was emphasized. After completing the second part of the research, the GPs filled out a 10-question questionnaire to confirm their understanding of the educational material. The research plan was approved by the Ethics Committee of the Faculty of Medicine, Osijek.

### Primary endpoint

The primary outcomes were i) the absolute number of first-line antibiotic prescriptions for uncomplicated cystitis in the same sample after the intervention, and ii) the absolute number of fluoroquinolone prescriptions.

The overall consumption was also evaluated according to the ESAC quality indicators for outpatient antibiotic prescribing. According to the ESAC criteria, an acceptable range of receiving a recommended antibiotic was between 80% and 100%, and for the fluoroquinolone below 5%.

### Statistical analysis

Summary statistics are expressed as absolute numbers and percentages, medians, quartiles, and interquartile ranges. The difference in the number of prescriptions before and after the intervention was assessed with a χ^2^ test. The difference in the prescription rates/100 patients before and after the intervention was tested with a Wilcoxon signed-rank test. Due to large disparities between individual physicians in the number of patients on the list, the absolute number of prescriptions was converted to rates per 100 patients, after which we analyzed the differences in medians of prescription rates before and after the intervention. To observe the minimum effect of 10% decrease in the number of prescriptions of the two dependent groups, with a significance level of 0.05 and a strength of 0.8, the minimum sample size required was 34 teams (GPower software, HHU, Düsseldorf, Germany). With a 10% increase, the optimal sample size was 38 teams. Since 42 physicians accepted to participate in the study, they were all included in the sample. Statistical analysis was performed by using the TIBCO Statistica, version 13.5.0.17 (TIBCO Software Inc., Palo Alto, CA, USA).

## RESULTS

Baseline characteristics of patients and GPs are presented in Table 1. The total number of patients on GPs’ lists was 67 547. Most of them were adults and the elderly (59 240 or 87.7%). There were only 747 children, because most preschool children in Croatia are treated by primary pediatricians, who were not included in the sample. Furthermore, there were no pregnant women in the sample because in Croatia they are treated by primary gynecologists. Most GPs were specialists in family medicine (64%). Physicians in the sample were equally represented in terms of place of work (59% urban vs 41% rural) and length of service.

**Table 1:** The characteristics of general practitioners (N = 42) involved in the study and their patients

Characteristics	N	%
**Patients**		
Sex		
male	31 214	46.21
female	36 333	53.78
Age (years)		
0-6	747	1.1
7-18	7570	11,2
19- 64	42 548	63
≥65	16 682	24.7
total	67 547	100
**General practitioners**		
Sex		
male	8	19
female	34	81
Completed medical specialty		
yes	27	64
no	15	36
Practice residence		
urban	25	59
rural	17	41
Years in practice		
<15	25	59
≥15	17	41

The total number of antibiotics prescribed for acute cystitis before and after the intervention is shown in Table 2. During the study period, 5317 antimicrobial prescriptions were issued for all UTIs combined (complicated and uncomplicated), of which 3851 (67.3%) were issued for uncomplicated cystitis (1717 before and 1864 after the intervention). A total of 1466 (32.7%) prescriptions were for complicated infections, pyelonephritis, and infections in male patients. In the second period, the total number of prescriptions for uncomplicated cystitis slightly increased (from 1717 to 1864 prescriptions, or 8.56%), mainly at the expense of first-line antibiotics (from 837 to 1015, or 21.2%).

**Table 2:** Total number of antibiotics and the number of first-line antibiotics for uncomplicated cystitis before and after the intervention

	No. (%) of antibiotics for uncomplicated UTIs^‡^	
	before intervention	after intervention
First-line*	837 (48.7)	1015 (54.4)
nitrofurantoin	477 (60)	636 (62.6)
fosfomycin	215 (25.7)	238 (23.4)
co-trimoxazole	145 (1.3)	141 (13.9)
Quinolones	332 (19.3)	310 (16.6)
Beta-lactams^†^	548 (32)	539 (30)
Total	1717	1864

*χ2 = 6.89, *P* = 0.03 for first-line antibiotics.

†co-amoxiclav, cephalexin, cefuroxime, cefixime, cefpodoxime.

‡χ2 = 11.92, *P* = 0.002 for all antibiotics.

Among the first-line antibiotics (nitrofurantoin, fosfomycin, and co-trimoxazole), the prescribing of nitrofurantoin increased by 33.3% (159 prescriptions), while that of fosfomycin and co-trimoxazole slightly decreased (Table 2). Changes in the number of prescriptions were also evident at the individual prescriber level. The prescribing of first-line drugs increased in 25 teams, while in 17 teams prescribing was either unchanged or slightly decreased.

Regardless the differences in the number of patients on the list (by an order of magnitude of almost 3), the prescription rate/100 patients of first-line antibiotics increased (difference in medians before-after 0.24/100), as did the prescription rate of nitrofurantoin (difference before-after 0.15/100) (Table 3).

**Table 3:** Differences in prescription rates/100 patients for uncomplicated cystitis before and after the intervention (Wilcoxon signed-rank test)

	Median before	Interquartile range before	Median after	Interquartile range after	Difference of medians	95% confidence interval	P
Total antibiotics	232	1.19	242.9	1.6	-17.5	0.03-0.39	0.014
First-line antibiotics	123	0.84	146.5	0.85	-23.5	0.12-0.40	0.0007
nitrofurantoin	68	0.6	83	0.92	-15	0.09-0.37	0.0005
fosfomycin	23.5	0.41	25.5	0.31	-2	-0.04 to -0.12	0.18
co-trimoxazole	18.5	0.19	19.5	0.23	-1	-0.07 to -0.05	0.39
Fluoroquinolones	45	0.52	36	0.54	9	-0.12 to -0.04	0.173

The prescription rate of first-line antibiotics/100 patients significantly increased in the period after the educational intervention (*P* < 0.001, Table 3). The prescription rate of fluoroqinoles decreased, but the difference did not reach significance.

Among the first-line antibiotics, only the prescription of nitrofurantoin significantly increased (*P* < 0.001) after the intervention. The prescription of fosfomycin (*P* = 0.18) and sulfamethoxazole-trimethoprim (*P* = 0.39) did not significantly increase. However, the observation that physicians started to prescribe fosfomycin to a slightly greater extent suggests a positive change in prescribing practices.

Before the intervention, 332 prescriptions of fluoroquinolones were issued, most of them for norfloxacin (269/332 or 81%), which is the only fluoroquinolone for uncomplicated cystitis recommended by the Croatian guidelines. After the intervention, the proportion remained the same (273/310 or 88%). Most physicians did not prescribe ciprofloxacin or prescribed it only 1-2 times during both study periods because according to the Croatian Health Insurance Institute its prescription is allowed only after a recommendation by a hospital specialist.

Prescription quality indicators were as follows: 100% for the criterion “proportion of women over 18 with cystitis who were prescribed an antibiotic for systemic use” – acceptable range 100%; 51.7% for the criterion “proportion of women over 18 years with cystitis who were prescribed the recommended (first-line) antibiotic”– acceptable range 80%-100%; and 17.9% for the criterion “proportion of women who were prescribed fluoroquinolone” – acceptable range 5%. The proportion of women over 18 years who were prescribed a first-line antibiotic (criterion “b”) was 48.7% before the intervention and 54.4% after the intervention. The proportion of patients who were prescribed fluoroquinolone (criterion “c”) was 19.3% before the intervention and 16.6% after the intervention.

## DISCUSSION

In this study, a short-term practice-based intervention resulted in an 8.5% increase (147 prescriptions) in absolute numbers in the use of first-line antibiotics for uncomplicated cystitis, while the use of fluoroquinolones was reduced by 6.6% (22 prescriptions). The increase in the prescriptions of the three first-line antimicrobials together and nitrofurantoin separately was significant, thus indicating an improvement of prescribing habits. The study is the first of its kind among family physicians in Croatia, where prescribing patterns are characterized by a high consumption of broad-spectrum antibiotics, particularly co-amoxiclav and cephalosporins, and a low consumption of narrow-spectrum antibiotics. Although total outpatient antibiotic consumption in Croatia decreased by approximately 2.9%: from 16.94 defined daily doses/1000 inhabitants/d (DDD TID) in 2019 to 14.05 DDD TID in 2020, probably due to the COVID-19 pandemic, the trend toward the use of broad-spectrum antibiotics continued to increase (21).

In the last few years, the use of fluoroquinolones and cefuroxime for the treatment of UTIs has increased, while the use of nitrofurantoin remained at only 0.83 DDD TID and that of fosfomycin at 0.08 DDD TID (9,21). Because the main objective of our intervention was only to increase the use of first-line antibiotics, guideline-based prescribing in the strict sense was not assessed. Nevertheless, the aims and the outcomes of the intervention are consistent with the recommendations of the national guidelines. The increase in the use of first-line antibiotics (8.56%) observed in this study is difficult to compare with the findings of similar outpatient intervention studies because of methodological differences (study design, outcome measures etc). Randomized trials conducted in primary care settings showed an increase in the prescription of recommended antibiotics together between 17% and 23% (22), and an increase in the use of recommended antibiotics from 55.7% to 82.7%, with a concomitant decrease in the use of non-recommended prescriptions (fluoroquinolones, cephalosporins, and co-amoxiclav) from 44.4% to 17.1% (23).

In our research, the prescription of beta-lactams (co-amoxiclav, cefuroxime) associated with diagnoses of acute uncomplicated cystitis remained unchanged. A probable explanation is that GPs in Croatia frequently prescribe broad-spectrum beta-lactams, especially co-amoxiclav, for other common infectious syndromes (respiratory, skin, and soft-tissue infections) (21), and simply translate this habit into the segment of UTIs. The overuse of co-amoxiclav in primary care has been addressed in a number of studies. An interventional research in Ireland reduced the use of co-amoxiclav from 33% to 10%, thus suggesting its overuse in both respiratory and UTIs (23). Coliform resistance decreased after a significant post-intervention reduction in the use of fluoroquinolones, cephalosporins, and co-amoxiclav in primary care (24), while *E. coli* resistance decreased from 37% to 11% after the reduction in the use of co-amoxiclav (from 33% to 12%) in a hospital setting (25).

Our assumption that a simple educational intervention could improve the prescribing practice by increasing the number of first-line antibiotic prescriptions, while simultaneously reducing the prescription of fluoroquinolones was only partially confirmed. The number of fluoroquinolone prescriptions decreased by only 22, while the number of nitrofurantoin, fosfomycin, and sulfamethoxazole-trimethoprim prescriptions combined significantly increased by 178.

The prescription rate of nitrofurantoin has shown the largest increase (33.3%). The significant increase in nitrofurantoin prescription could be attributed to the adoption of specific educational messages by the majority of GPs in the sample. However, despite the significant increase, nitrofurantoin prescription accounted for only a third of the total consumption in acute cystitis, whereas broad-spectrum agents (fluoroquinolones and beta-lactams) accounted for more than a half. One of the reasons for the suboptimal use of nitrofurantoin, despite the key messages in educational intervention, has probably been the prescribers' inertia, or the misconception that broad-spectrum antibiotics are more reliable. Another reason may be possible therapeutic failures due to sub-dosing. Nitrofurantoin is available in Croatia only in the macrocrystalline form, while in some countries it is available in the macrocrystalline monohydrate form (with modified release) with a dosage of 100 mg every 12 hours. Current national guidelines recommend a dosage of 2 × 100 mg (or 3 × 100 mg in the latest update) (26), which is by no means sufficient for a macrocrystalline preparation with an elimination half-life of 0.5-1 hour to maintain effective bactericidal concentrations in urine. Therefore, it should be taken strictly every 6 hours 50-100 mg, but that information is missing in the guidelines.

Nitrofurantoin is increasingly used in both adult men and the elderly, although it has not been previously recommended for these groups (27), and is also effective in catheter-associated infections. Due to the increasing prevalence of multidrug-resistant *E. coli* in the community (4), nitrofurantoin will continue to play a dominant role in the treatment of lower UTIs because of its high efficacy against resistant pathogens (12). Among other first-line antibiotics, the prescriptions of fosfomycin increased by 10.7% (23 prescriptions), while co-trimoxazole prescriptions decreased by 4, although the difference was not significant.

Reduction in the use of fluoroquinolones and cephalosporins is of crucial importance in the control of antibiotic resistance (28). Interventional studies have achieved variable, mostly positive outcomes (29). Most of them used a passive educational strategy of one-way delivery of key messages, with no pronounced differences between low-intensity and high-intensity education (30). In a systematic review of interventions in primary care, the authors concluded that interventions that used printed educational materials generally failed to produce any serious or lasting qualitative changes in prescribing patterns (31). Although passive educational methods (didactic lectures, printed educational material) that do not encourage discussion, had a limited effect (31), some studies demonstrated their excellent performance (32).

The learning process in prescribing antimicrobials in primary care is relatively long-term and subject to multiple influences from a variety of sources – literature, guidelines, recommendations from specialists (feedback from referrals) and from peers (33). GPs in our study demonstrated that they could change their prescribing habits in a relatively short time. A single educational article that emphasized key messages was relatively effective in refreshing knowledge and acceptance of more appropriate prescribing practices. Despite the feedback (sending responses from the questionnaire), the intervention had an almost exclusively passive component of knowledge transfer, with no information exchange with the researchers or between neighboring family practices.

When it comes to the ESAC quality indicators, the indicator “b” increased by 5.7%, but still did not reach the lower end of the acceptable range (80%-100%), while the proportion of fluoroquinolones (17.9%) was well above the acceptable range (0%-5%), similar to the findings of a recent Hungarian study (34).

A relatively high consumption of fluoroquinolones (24%) was also reported in a Swedish study in 2013, but, in contrast to our results, with the consumption of nitrofurantoin and pivmecilinam at 69% (35). In a recent Swiss study, the prescription of first-line antibiotics was 84.7%, and that of fluoroquinolones was 14.5% (36).

The main advantage of our research is the methodology of data collection: collection of written prescriptions issued in real time and during routine work in offices, rather than the collection of data on dispensed antibiotics. The main limitation is that we could not record delayed prescriptions, a particularly specific and suitable tool in antimicrobial pharmacotherapy in family practice.

In conclusion, a relatively short-term intervention changed antimicrobial prescribing in a positive direction. Educational intervention could further be enhanced by providing feedback to GPs on the quality of their prescribing patterns. The feedback from physicians would also be helpful in creating shorter but more meaningful educational articles. Such problem-oriented materials are probably better accepted than voluminous guidelines.
